# FlashFry: a fast and flexible tool for large-scale CRISPR target design

**DOI:** 10.1186/s12915-018-0545-0

**Published:** 2018-07-05

**Authors:** Aaron McKenna, Jay Shendure

**Affiliations:** 1 Department of Genome Sciences, University of Washington, Seattle, WA USA; 2 Howard Hughes Medical Institute, Seattle, WA USA

**Keywords:** CRISPR, Guide library, Cas9, Cpf1, Off-target, On-target, Deletion scan

## Abstract

**Background:**

Genome-wide knockout studies, noncoding deletion scans, and other large-scale studies require a simple and lightweight framework that can quickly discover and score thousands of candidate CRISPR guides targeting an arbitrary DNA sequence. While several CRISPR web applications exist, there is a need for a high-throughput tool to rapidly discover and process hundreds of thousands of CRISPR targets.

**Results:**

Here, we introduce FlashFry, a fast and flexible command-line tool for characterizing large numbers of CRISPR target sequences. With FlashFry, users can specify an unconstrained number of mismatches to putative off-targets, richly annotate discovered sites, and tag potential guides with commonly used on-target and off-target scoring metrics. FlashFry runs at speeds comparable to commonly used genome-wide sequence aligners, and output is provided as an easy-to-manipulate text file.

**Conclusions:**

FlashFry is a fast and convenient command-line tool to discover and score CRISPR targets within large DNA sequences.

## Background

The CRISPR prokaryotic immune system has transformed genome engineering. As typically used, CRISPR proteins are directed to create double-stranded DNA breaks at location(s) in a genome matching a specified guide sequence [1]. These double-stranded breaks are commonly repaired by a non-homologous end joining (NHEJ) pathway, which can leave small insertions or deletions (indels) at the genomic target site. These site-specific, targeted indels can be used to perturb endogenous gene function [2], encode information [3], or characterize the function of genomic sequence [4–6].

Although CRISPR editing is specific [7], not all guides function with the same efficiency or specificity. For instance, double-stranded breaks can occur at genomic locations (“targets”) that are an imperfect match to the supplied guide sequence (termed “off-targets”). To reduce the chance of such unintended genome editing, guide sequences can be chosen that contain less overlap with all possible alternative targets in the genome. The importance of specific differences in the guide sequence, the genomic location and chromatin environment of the target, and the method of guide delivery all appear to affect the distribution and rate of this off-target cutting [8].

To help users choose both specific and active guide sequences, the community has created a large number of CRISPR target selection tools, most of which are made available as web applications [8–10]. Such tools are convenient for researchers screening a small set of guides or scanning a single genomic locus like an exon. Unfortunately, these tools require batched queries for large sets, which makes it more challenging to scan loci or whole genomes for guides. Additionally, some guide screening tools rely upon genome-wide alignment tools to generate putative off-target lists for each guide. For practical reasons, these aligners are generally designed to quickly discover only the most similar sequences with a limited number of mismatches in comparison to the guide (typically ≤3), whereas experimental efforts have shown activity at off-target sequences containing upwards of six mismatches to the guide [11]. Some of these tools also miss a subset of potential off-targets altogether, regardless of the mismatch distance [12]. Lastly, end users may need to design guides to non-model organisms, engineered DNA sequences, or genomic sequences that contain single nucleotide variants [13]. To address these issues, we have created FlashFry, a command-line tool for discovery and characterization of CRISPR guide sequences from arbitrary genomic regions.

## Implementation

### Database creation

**Fig. 1:**
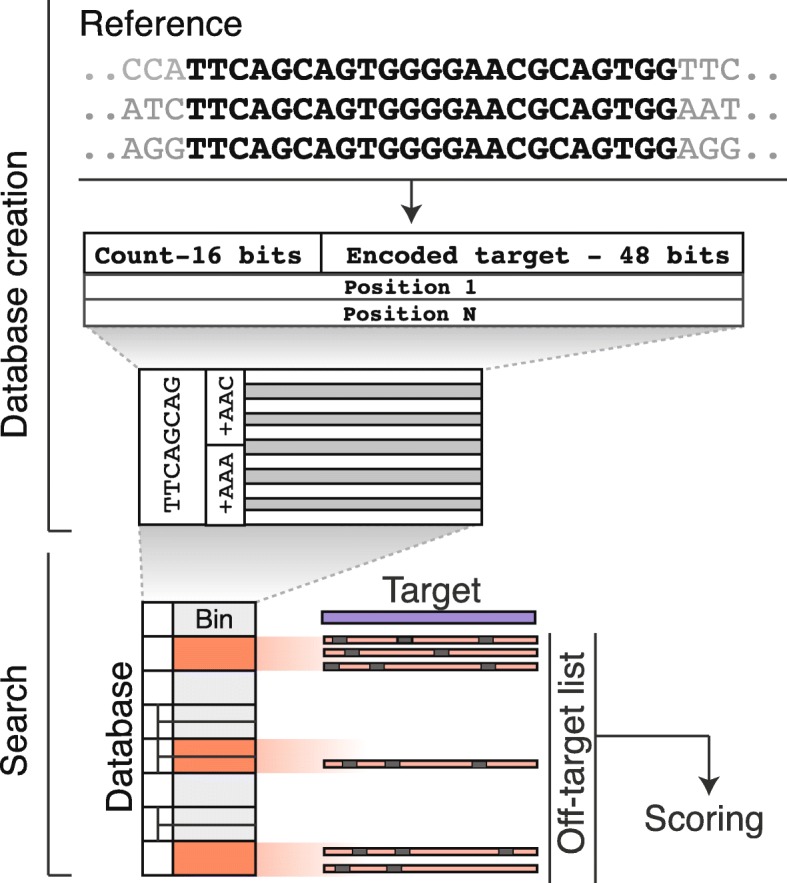
Discovery and scoring of CRISPR target sites. FlashFry schematic. The genome of interest is scanned for targets that match the PAM of the specified CRISPR enzyme. These genomic targets are then aggregated and bit-encoded into a database of compressed bins, sorted by their prefix. This database can then be searched by comparing the prefix of a candidate target against the prefix of the bins, and bins within the allowed mismatch (orange) can be examined for individual off-targets in the genome. The resulting off-target list is aggregated and used by various scoring metrics

FlashFry generates a block-compressed binary database of all potential target sequences within a given reference sequence. This FASTA-formatted sequence can be the genome of a canonical model organism, the transcriptome of a new species, or a custom-built reference that integrates strain or cell-line-specific variants. This database is generated for a specific CRISPR enzyme and protospacer adjacency motif (PAM) combination and can be constructed in approximately an hour on a standard computer (Table 1). In this database, sequences that contain the required CRISPR PAM sequence are organized into sorted prefix-bins (Fig. 1). This prefix length can be specified at runtime, with larger bins being automatically sub-indexed to reduce lookup times. The resulting data structure can then be quickly searched by comparing a target sequence’s prefix against the bin’s sequence. To save space and to further improve search times, target sequences and their number of occurrences within the genome are stored as a 64 bit-encoded value within each bin. Each target sequence is then followed by additional binary-encoded values for each position within the genome. These bins are compressed using the HTSJDK library [14], and an index is created for each bin’s offset within this file.

**Table 1:** A sample of computational times (h:m:s) required to build a FlashFry database for versions of the *Caenorhabditis elegans*, human, mouse, and *Drosophila melanogaster* genomes for common CRISPR enzymes. All timing analyses were run with default FlashFry parameters on an Amazon r4.large instance, limited to 8 GB of memory limited and one CPU core

Genome	CRISPR/Cas9 (NGG)	CRISPR/Cas9 (NGG/NAG)	Cpf1 (TTTN)
*Caenorhabditis elegans*—235	0:1:28	0:2:55	0:2:13
Human—hg38	1:15:15	2:42:27	0:56:23
Mouse—mm10	1:02:13	2:21:11	0:43:13
*Drosophila melanogaster*—BDGP6	0:2:38	0:5:01	0:2:14

### Search

Given the inherent inefficiencies of high-mismatch searches, FlashFry uses a filtering approach to find candidate off-targets. It does so by precomputing a traversal over target bins with less than *k* mismatches to each guide in the candidate (such filtering approaches are well reviewed in Navarro et al. [15]). Each target sequence is compared to the prefix of every bin, and only those bins with less than or equal to *k* mismatches are recorded as potential search locations. The list of visited bins then aggregated for all targets, these bins are loaded sequentially, and the genomic target sites within are compared against each target with a matching prefix. When a large number of bins are to be searched (> 95% of all bins), which is common with large guide screens or with a high *k* mismatch threshold, FlashFry will instead search the full database to avoid the overhead cost of disk seeks.

To further reduce search times, FlashFry uses bit parallelism when determining mismatches between the binary-encoded target and candidate genomic matches [15]. Bit parallelism uses bit-shifting and optimized processor instructions to compare the binary representations of nucleotide strings in a small number of compute operations, avoiding a character by character string comparison. FlashFry is currently compatible with target sequences up to 24 bases in length, although it could be expanded to longer target sequences, as our bin storage approach allows recovery of the prefix of a target sequence (allowing target sequences of length 24 plus the prefix length). Lastly, off-target discovery is halted for candidate guides that have exceeded a user-defined number of off-target hits, saving compute time by eliminating poor candidates early from the putative target pool. This off-target limit can be set by the user, defaulting to 2000 off-targets.

**Fig. 2:**
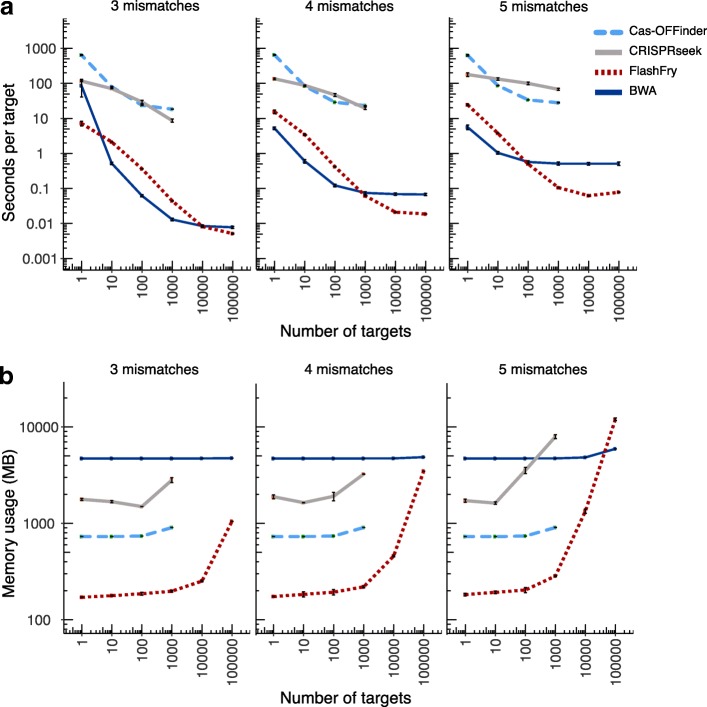
Comparison of the runtimes and memory usage of common CRISPR target discovery tools over an increasing number of targets and permitted mismatches. Five random CRISPR guide sets were run for each target-count (*x*-axis) and permitted mismatch level (*y*-axis). Plotted are the mean runtime with standard deviation bars for each set of replicates. **a** Running time per sequence for increasing numbers of target sites and **b** their corresponding memory usage. FlashFry benefits from aggregating all guide-to-genome comparisons in one pass of the database, matching BWA’s performance at hundreds of targets for five mismatches, and thousands of targets at four mismatches. Only BWA and FlashFry were run for the 10,000 and 100,000 target searches

We then compared the runtime and memory usage of FlashFry to the commonly used CRISPR characterization tools Cas-OFFinder and CRISPRseek [16, 17]. We also included BWA in this comparison, which is used as a backend search tool for some CRISPR web applications [8, 18]. For all tools, per-target search times decrease with the number of targets and the allowed number of mismatches (Fig. 2a). FlashFy and BWA run approximately two to three orders of magnitude faster than the conventional CRISPR target discovery tools. FlashFry outperforms BWA for larger target sets and at higher allowed mismatches between the candidate guide and its genomic targets, though at the cost of higher memory usage for very large target sets (Fig. 2a, b).

### Guide characterization and scoring

The goal of a typical biologist is to pick a subset of highly active and specific CRISPR guide sequences from a full list of candidate targets within a region of interest. Therefore, FlashFry includes many commonly used scoring approaches, including off-target metrics such as cutting-frequency determination (CFD) [12], and the Hsu et al. scoring scheme [19], and both the Moreno-Mateos and Vejnar et al. (CRISPRScan) and the Doench et al. 2014 on-target metrics [11, 16]. We have also included a set of basic design criteria filters, including flags for high and low GC content, warnings for poly-thymine tracts (which can prematurely terminate polymerase III transcription), and putative targets that have reciprocal off-targets within the region of interest (potentially leading to deletions of the intervening sequence). Lastly, regions can be annotated with information from external BED files, which may be useful for highlighting repetitive sequences or regulatory regions.

## Results

To demonstrate how FlashFry can be used to create a CRISPR library, we targeted all of the genes within the cancer gene census, a curated list of commonly mutated genes in cancer patients [20]. We retrieved genomic sequences corresponding to all 10,808 exons; allowing only NGG PAMs, FlashFly discovered off-targets for 443,296 candidate SpCas9 targets in 250 min (0.03 s per guide) on an Amazon r4.xlarge instance. We then excluded 16,736 target sequences that exceeded the maximum off-target count (> = 2000), and 66,637 that were annotated as having extreme GC content or runs of thymines that can limit expression in certain plasmid delivery systems. The resulting targets were annotated with the CRISPRScan on-target and Hsu et al. off-target scores and aggregated into a combined rank score using the Schulze method (see the “Methods” section). The resulting table can then be used to generate a diverse library of CRISPR guides for a perturbation screen. For instance, over 99% of gene bodies have two targets with a Hsu et al. score over 75 and a CRISPRScan score over 0.50. At the exon level, 74 and 71% have two targets both with scores above 75 and 0.50 respectively, allowing for redundant knockout of most exons. These filtered candidate libraries can be easily processed into the format required by a wide variety of custom microarray manufacturers.

## Conclusions

The needs of genome-wide knockout studies, noncoding deletion scans, and other large-scale studies or method development projects are unfortunately not well-met by the abundant CRISPR web applications. FlashFry, an efficient and flexible toolset, fills this void and can be used to rapidly discover and characterize tens to hundreds of thousands of guides from an arbitrary sequence quickly and with a relatively low memory footprint. For method developers, we also expose a simple interface for implementing additional scoring schemes, given the sequence context of a target and its off-target hits within the genome. FlashFry has no system dependencies outside of the JVM and avoids many of the configuration pitfalls and complexity of tools that rely on genome aligners to discover off-target sequences. Documentation, code, and tutorials are available on the FlashFry GitHub website.

## Methods

### Software availability

FlashFry is written in Scala and bundled as a single stand-alone Jar file, easily run on any system with an installed Java virtual machine (JVM). The tool is freely licensed under version 3 of the GPL, and code, documentation, and tutorials are available on the GitHub page: https://aaronmck.github.io/FlashFry/

### Database generation times

We timed FlashFry’s database creation on an Amazon’s Elastic Compute Cloud r4.large instance type using an SSD filesystem. FlashFry was limited to one CPU with the ‘taskset -c 0’ command and 8GB of memory using the java “-Xmx8g” argument.

### Off-target tool comparisons

Runtime and memory comparisons were run on Amazon’s Elastic Compute Cloud using the r4.large instance type with an Intel Xeon CPU at 2.30 GHz. Each machine instance was setup using a Docker configuration file, which is provided within our GitHub codebase. Each tool was limited to a single processor with the “taskset” Linux command, and compute and memory usage were recorded with the “time -v” command. The full pipeline is available in the GitHub repository, along with the timing results of individual runs. Each guide-count and permitted mismatch (3,4, and 5) value was replicated five times using the human HG38 reference. Guide counts of 1, 10, 100, and 1000 were run for all tools. These random guides were generated using FlashFry’s “random” analysis module with the “—onlyUnidirectional” flag set to ensure a single candidate per sequence. Additionally 10,000 and 100,000 guide iterations were run for FlashFry and BWA, but were not run with Cas-OFFinder and CRISPRseek for practical reasons. Individual tool configurations are detailed below.

### FlashFry

FlashFry version 1.8.1 was run in discovery mode with java option “-Xmx8g” for 1–1000 target runs, and “-Xmx15g” for 10,000 and 100,000 target searches. Mismatches were set with the “--maxMismatch” command, and defaults were used for all remaining parameters.

### BWA

BWA runtime includes the initial alignment step (aln) and mapping to genomic coordinates (samse). BWA aln was run with parameters taken from Haeussler et al. [8]: *aln -o 0 -m 20000000 -n <mismatch_count> -k <mismatch_count> -N -l 20 <humanRef>*, using BWA commit tag e624290ad42f6c1deea87332337b08302faece48 from the following repository: https://github.com/lh3/bwa.

### Cas-OFFinder

A custom script was used to convert the random target FASTA file into the Cas-OFFinder input with the appropriate mismatch setting, using the 20(N)NGG search string. This conversion time was not towards Cas-OFFinder’s runtime or memory usage. Cas-OFFinder requires a custom Linux kernel driver supporting OpenCL to be installed on the machine, and our Docker instance pre-configures Intel’s OpenCL version 2017_7.0.0.2568_x64. Cas-OFFinder was then run using the CPU “C” option against the input file.

### CRISPRseek

We ran CRISPRseek using a custom R script and the “Rscript” command-line tool (the associated code is available in our GitHub repository). Timing data includes loading the relevant libraries and resources, executing the off-target search, and as it was impossible to separate discovery of off-targets and scoring, the scoring of guides.

### Cancer gene census calculations

All processing was done on a standard Amazon AWS r4.xlarge compute instance with an Intel Xeon CPU at 2.30 GHz. The cancer gene census (CGC) dataset, version 83 was downloaded from the CGC portal [20]. Intervals were generated using custom Scala scripts capturing the RefSeq exonic sequence of each gene using the model with the largest number of exons, adding 10 bases up and downstream. The corresponding genomic sequences were extracted from the human HG19 genome using Picard’s ExtractSequences (https://broadinstitute.github.io/picard/). Sites were then discovered and scored using FlashFry with a maximum of four mismatches to off-targets, and a maximum of 2000 off-target sequences per candidate. Off-target scoring was run with 30 GB of memory, taking 15,024.70 s. The Hsu et al. off-target scoring scheme [19] and the Moreno-Mateos and Vejnar et al. [10] on-target metric were run against the 426,560 sites, and an aggregate ranking was produced by supplying FlashFry the “rank” scoring option. The rank option will produce a rank-ordered assignment for each target based on the median rank of individual scores and will additionally use the Schulze method to rank the top 1000 targets [21]. Lastly, the best and second-best hits per individual exon and gene were calculated with Python and R scripts, available in the GitHub repository.

## Availability and requirements

Project name: FlashFy

Project home page: http://aaronmck.github.io/FlashFry/

Operating system(s): Platform independent

Programming language: Scala/Java/JVM

Other requirements: Java 1.8 or higher

License: GNU GPL v3

Any restrictions to use by non-academics: None
